# Cultivation of recombinant *Aspergillus niger* strains on dairy whey as a carbohydrate source

**DOI:** 10.1093/jimb/kuae007

**Published:** 2024-01-31

**Authors:** Teagan C Crament, Kayline Arendsen, Shaunita H Rose, Trudy Jansen

**Affiliations:** Department of Microbiology, Stellenbosch University, Stellenbosch 7600, South Africa; Department of Microbiology, Stellenbosch University, Stellenbosch 7600, South Africa; Department of Microbiology, Stellenbosch University, Stellenbosch 7600, South Africa; Department of Microbiology, Stellenbosch University, Stellenbosch 7600, South Africa

**Keywords:** *Aspergillus niger*, Endoglucanase, Recombinant, Lactose, Whey

## Abstract

Agricultural waste valorisation provides a sustainable solution to waste management, and combining waste utilisation with commodity production allows for responsible production processes. Recombinant *Aspergillus niger* D15 strains expressing fungal endoglucanases (*Trichoderma reesei eg1* and *eg2* and *Aspergillus carneus aceg*) were evaluated for their ability to utilise lactose as a carbon source to determine whether dairy waste could be used as a feedstock for enzyme production. The recombinant *A. niger* D15[eg1]PyrG, D15[eg2]PyrG, and D15[aceg]PyrG strains produced maximum endoglucanase activities of 34, 54, and 34 U/mL, respectively, on lactose and 23, 27, and 22 U/mL, respectively, on whey. The *A. niger* D15[eg2]PyrG strain was used to optimise the whey medium. Maximum endoglucanase activity of 46 U/mL was produced on 10% whey medium containing 0.6% NaNO_3_. The results obtained indicate that dairy whey can be utilised as a feedstock for recombinant enzyme production. However, variations in enzyme activities were observed and require further investigation.

## Graphical Abstract

**Figure:**
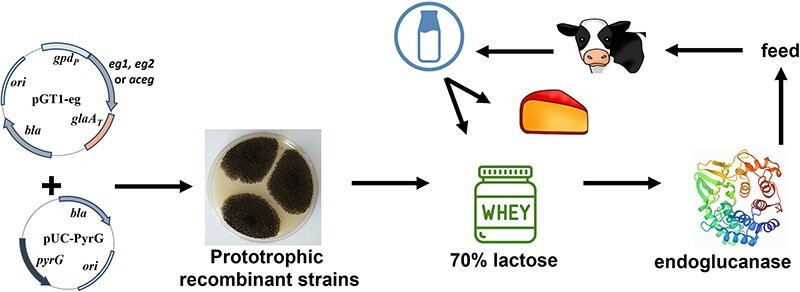

## Introduction

The rise in the world population requires an increase in the food supply without disruption of the existing food-producing practices. The primary obstacles that need to be addressed are twofold: First, to enhance food production by implementing strategies to minimise agricultural product losses, specifically reducing unnecessary wastage. Second, there is a need to reduce waste generation across various processes related to food production and consumption. Approximately 906 million tonnes of milk were produced globally in 2020, making it one of the most widely consumed beverages in the world (FAO, 2021). Milk is used to produce a variety of food products, such as butter, ice cream, cheese, etc. Whey is an abundant and inexpensive by-product of the cheese industry, with approximately 1 kg of manufactured cheese generating 9 L of whey (Fernández-Gutiérrez et al., 2017). Sweet whey has a pH of 6–7 and is produced when chymosin is used for enzymatic coagulation. Acid whey is a by-product of acidified milk coagulation and has a pH of less than 5 (Fernández-Gutiérrez et al., 2017). Whey contains 93–95% (w/w) water, 50% milk nutrients, and 60–70 g/L solids (Fernández-Gutiérrez et al., 2017). The dry matter fraction contains 66–77% (w/w) lactose, 8–15% (w/w) proteins, and 7–15% (w/w) minerals and vitamins. Whey is an environmental pollutant due to the high biochemical (30–50 g/L) and chemical (60–80 g/L) oxygen demand resulting from the high lactose (39–45 g/L) and protein (9–14 g/L) content (Fernández-Gutiérrez et al., 2017; Lech, 2020). The biotechnological challenge is (1) to decrease the waste produced in the agriculture industry without disruption of the existing food-producing practices and (2) to minimise the loss of agricultural products through unnecessary waste production. The whey proteins are highly desirable due to their health benefits and are recovered using micro- and ultra-filtration methods, leaving the sterile whey permeate for lactose recovery (Lech, 2020). These are expensive technologies resulting in valuable products, both of which are not feasible options for developing countries. Whey is supplied to local pig farms as an animal feed additive; however, the whey supply outweighs the demand. Proper waste treatment of huge volumes is costly, and untreated whey is discarded in sewer systems and rivers, which affects ecosystems and leads to the loss of biodiversity (Fernández-Gutiérrez et al., 2017).

A large market exists for cellulases, ranging from biofuel production to the food and feed industries. As with all industrial processes, the cost of the feedstock for enzyme production is a critically important determinant of the profit margin of the enzyme industry. Therefore, using a waste product as feedstock for a biorefinery can lower the production cost. Since whey is readily available at a low cost in developing countries, it is fitting to investigate the possible use of whey as a feedstock, with the additional advantage of having a positive environmental impact.

The use of *Aspergillus* spp. for the large-scale production of industrially relevant enzymes is well documented (Cairns et al., 2018; Li et al., 2020). Specific features that favour *Aspergillus* spp. for industrial use include its GRAS (**g**enerally **r**egarded **a**s **s**afe) status, high secretion capacity, proper folding of proteins without hyperglycosylation and rapid growth on inexpensive media. *Aspergillus niger* for example, is used for the large-scale production of enzymes such as pectinases, β-galactosidases, cellulases, etc., of which GlaA glucoamylase is produced at titres of 30 g/L (Cairns et al., 2021). The genus also produces a wide range of organic acids, with some strains producing up to 200 g/L citric acid (Cairns et al., 2021). *A. niger* can utilise lactose as a carbon source due to the expression and secretion of five β-galactosidases (EC 3.2.1.23) that hydrolyse the lactose to glucose and galactose (Niu et al., 2017). The ability to metabolise lactose makes the genus *Aspergillus* the ideal organism for cultivation on whey with simultaneous production of recombinant enzymes for the food or animal feed industry.

The study was designed in alignment with the goals for sustainable development. Instead of disposing whey into the environment, which could pose significant environmental risks and lead to nutrient loss, the research aimed to explore an economically viable approach for managing whey waste. Additionally, the study sought to create a valuable resource for the agriculture or food industry. To achieve these goals, the research focused on examining the constitutive expression of the endoglucanase-encoding genes from *Trichoderma reesei* (*eg1* and *eg2*) and *Aspergillus carneus* (*aceg1*) in *A. niger* on lactose using a chemically defined and whey medium.

## Materials and Methods

### Strains

The relevant genotypes of the filamentous fungal strains and vectors used in this study are summarized in Table 1. The *A. niger* D15#26 strain is a protease-deficient strain that does not acidify the growth media (Gordon et al., 2000; Wiebe et al., 2001), resulting in high protein titres with diminished degradation by natively produced acid proteases. The fungal strains were constructed using spheroplasts generated by lysing enzymes (Punt & van den Hondel, 1992). The construction of the *A. niger* D15[GT], D15[eg1] and D15[eg2] and D15[aceg1] strains were previously described in Rose & van Zyl (2002, 2008). The endoglucanase encoding genes are expressed under the control of the constitutive *A. niger* glyceraldehyde 3-phosphate (*gpd*) promoter and *Aspergillus awamori* glucoamylase (*glaA*) terminator. The pUC-PyrG vector was introduced and integrated into the genomes of *A. niger* D15[GT], D15[eg1], D15[eg2], D15[aceg1], resulting in the prototrophic *A. niger* D15[GT]PyrG, D15[eg1]PyrG, D15[eg2]PyrG, and D15[aceg]PyrG strains (Table 1). The *A. niger* D15[GT]PyrG strain served as a reference strain. The *A. niger* D15[eg1] and D15[eg2] and D15[aceg1] strains were selected based on the pH optima of the recombinant endoglucanases (Table 2).

**Table 1.** The Relevant Genotype and Sources of the Strains and Vector

Strains	Genotype	Reference
***E. coli***		
*E. coli* DH5*α*	*supE44 _lacU169 (ϕ80lacZ&M15) hsdR17 recA1 endA1 gyrA96 thi*-*1 relA1*	Sambrook et al. (1989)
DH5*α*[pUC-PyrG]	*bla PyrG*	Ling et al. (2013)
***A. niger***		
D15#26	*cspA1, pyrG1, prtT13, phmA* (nonacidifying)	Gordon et al. (2000)
D15[GT]	*amdS gpd*_P_ *-glaA*_T_	Rose and van Zyl (2002, 2008)
D15[eg1]	*amdS gpd*_P_ *egI-glaA*_T_	Rose and van Zyl (2002)
D15[eg2]	*amdS gpd*_P_ *-eg2-glaA*_T_	Rose and van Zyl (2008)
D15[acegI]	*amdS gpd*_P_ *-aceg-glaA*_T_	Rose and van Zyl (2008)
D15[GT]PyrG	*amdS PyrG gpd*_P_ *-glaA*_T_	This study
D15[eg1]PyrG	*amdS PyrG gpd*_P_ *-egI-glaA*_T_	This study
D15[eg2]PyrG	*amdS PyrG gpd*_P_ *-eg2-glaA*_T_	Rose et al. (2018)
D15[aceg]PyrG	*amdS PyrG gpd*_P_ *-aceg-glaA*_T_	This study
**Plasmid**		
pUC-PyrG	*bla PyrG*	Ling et al. (2013)

**Table 2.** The Relevant Information of the Endoglucanases

Gene	Enzyme	Accession nr	Predicted mass (kDa) unglycosylated	pH optimum^1^
*Trichoderma reesei eg1*	Eg1	M15665	48	5
*Trichoderma reesei eg2*	Eg2	M19373	44	4.8
*Aspergillus carneus aceg* ^2^	Aceg	AJ224451	26	4.2

^1^Rose and van Zyl (2008).

^2^ *aceg* has a 100% DNA sequence homology to the *A. niger egA*.

### Cultivation Medium and Conditions

All reagents and media components were supplied by Merck (Darmstadt, Germany) unless stated otherwise. The *Escherichia coli* DH5*α* strain (Takara Bio Inc., Kusatsu, Shiga, Japan) was used for plasmid propagation with transformants maintained and selected on Luria Bertani agar (5 g/L yeast extract, 10 g/L tryptone, 10 g/L NaCl, and 20 g/L agar) containing 100 μg/mL ampicillin. The *E. coli* DH5α[pUC-PyrG] strain was cultured at 37°C in Terrific Broth (24 g/L yeast extract, 12 g/L tryptone, 4 mL/L glycerol, and 0.1 M potassium phosphate buffer) containing 100 mg/L ampicillin.

Auxotrophic fungal strains were cultivated in minimal media (MM) containing 5 g/L yeast extract, 0.4 g/L MgSO_4_.7H_2_O, 2 g/L casamino acids, 20 mL 50× AspA (300 g/L NaNO_3_, 26 g/L KCl, 76 g/L KH_2_PO_4_, pH 6), 0.01 M uridine, 10 g/L glucose, and trace elements before transformation (Punt & van den Hondel, 1992; Rose & van Zyl, 2002). Prototrophic transformants were selected on a minimal medium lacking uridine. Prototrophic strains were routinely cultivated in a double-strength minimal medium (2×MM) containing 100 g/L lactose or glucose as a carbohydrate source. The dried soy whey (Avonlac^®^ 282, Glanbia Nutritionals, www.glanbianutritionals.com, and Nandrea Health Products, Oudtshoorn, South Africa) was prepared as a 10% w/v solution containing 100 mg/mL erythromycin. Liquid bovine whey (Roulou, Stellenbosch, SA) was prepared as a 10% v/v solution containing 100 mg/mL erythromycin. All cultivations took place at 30°C in 125 mL flasks containing 20 mL of medium, with agitation at 200 rpm.

### Qualitative Plate Assays

Transformants were screened on Ostazin brilliant red hydroxyethyl cellulose (OBR-HEC) agar plates (6.7 g/L yeast nitrogen base without amino acids [BD-Diagnostic Systems, Sparks, MD], 100 g/L glucose, 10 g/L OBR-HEC and 20 g/L agar). Spores were transferred to the OBR-HEC, agar plates, and the development of the clearing zone was monitored for 24 hr.

### Quantitative Liquid Assays

Volumetric endoglucanase activity was determined using the reducing sugar assay (Miller, 1959) and 1% CMC (in 0.05 M citrate phosphate buffer, pH 5.0) as substrate. Recombinant fungal strains were cultivated aerobically in 125 mL Erlenmeyer flasks containing 20 mL 2×MM at 30°C for 7 days at 200 rpm. Supernatant samples were collected at 24 hr intervals. All assays were conducted in triplicate, and the absorbance was measured at 540 nm using the xMark Microplate Spectrophotometer (Biorad, Hercules, CA).

### SDS-PAGE

Recombinant fungal strains were cultivated in 20 mL 2×MM medium containing 10% glucose in 125 mL Erlenmeyer flasks for 48 hr at 30°C and 200 rpm. Crude supernatant protein samples (15 mL) were separated using an 8% polyacrylamide separation gel (Laemmli, 1970; Sambrook et al., 1989) at 100 V for 90 min. Proteins were visualised using the silver staining method (O'Connell and Stults, 1997). The prestained PageRuler^TM^ (Thermo Fischer Scientific) was used for size estimation of the recombinant endoglucanases.

## Results and Discussion

*Aspergillus niger* is a desirable host for large-scale production of proteins or enzymes due to the ability to secrete large titres (grams per litre) of proteins or enzymes (Cairns et al., 2018; Li et al., 2020). The production of a diverse range of proteases (especially acid proteases) remains a hurdle in enzyme production since medium acidification naturally occurs during cultivation.

**Fig. 1.**
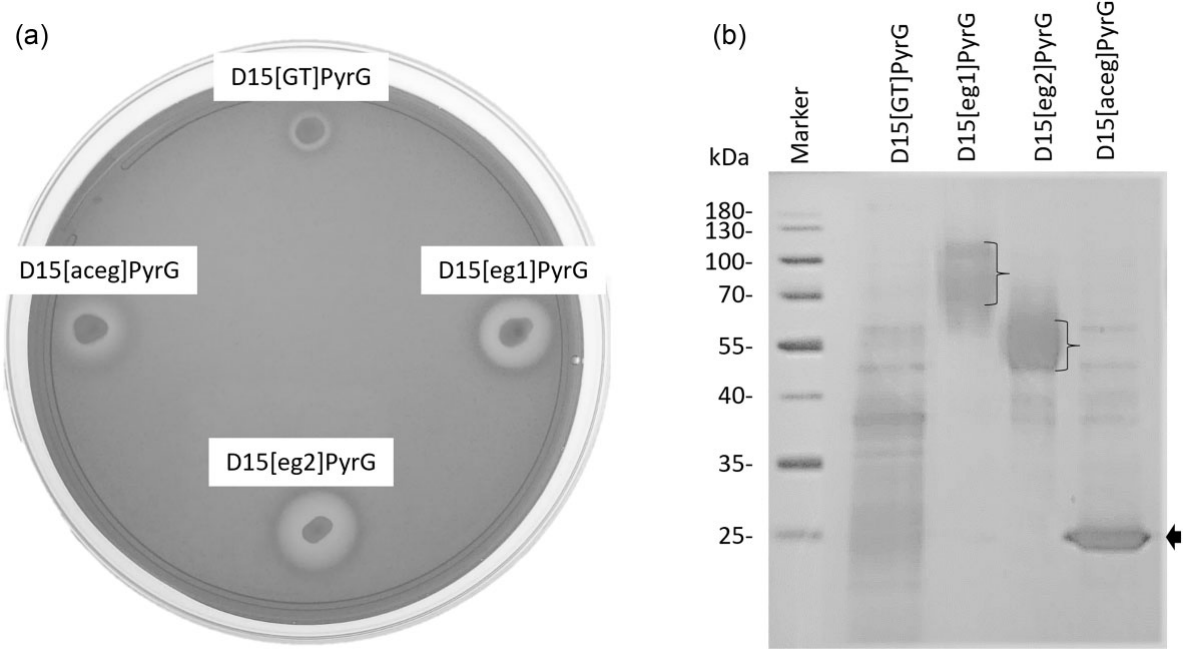
Endoglucanase expression in the recombinant *A. niger* strains. The *A. niger* D15[GT]PyrG, D15[egI]PyrG, D15[eg2]PyrG, and D15[aceg]PyrG strains were cultivated on OBR-HEC agar plates for 24 hr at 30°C. (a) The clearing zone around the strains is indicative of endoglucanase activity. (b) SDS-PAGE analysis of the extracellular proteins produced by recombinant strains cultivated aerobically for 48 hr in 2 × MM containing 10% glucose. The molecular mass marker with the sizes (kDa) is depicted in the far-left lane. *A. niger* D15[GT]PyrG is the reference strain and serves as the negative control.

Spores from the *A. niger* D15[GT]PyrG, D15[eg1]PyrG, D15[eg2]PyrG, and D15[aceg]PyrG strains were transferred to OBR-HEC agar plates containing 10% (w/v) glucose. The *A. niger* D15[eg1]PyrG, D15[eg2]PyrG, and D15[aceg]PyrG strains started to develop a clearing zone within 5 hr of germination, which is indicative of extracellular endoglucanase activity (Fig. 1a). The use of the constitutive *gpd* promoter resulted in the expression of the genes in the presence of glucose. The *A. niger* D15[GT]PyrG only started to produce a zone after approximately 24 hr. The depletion of glucose in the medium surrounding the *A. niger* D15[GT]PyrG strain results in the relief of catabolite repression of the native endoglucanases, leading to delayed zone formation.

The *A. niger* D15[GT]PyrG, D15[egI]PyrG, D15[eg2]PyrG, and D15[aceg]PyrG strains were cultivated in 2×MM containing 10% glucose for 48 hr. Supernatant samples were obtained and used directly for SDS-PAGE without further purification. The Eg1, Eg2, and AcEg recombinant enzymes are the prominent protein species in the supernatant samples obtained from the *A. niger* D15[egI]PyrG, D15[eg2]PyrG, and D15[aceg]PyrG cultures, respectively (Fig. 1b), which supports the finding of Rose and van Zyl (2002). The Eg1 and Eg2 are produced as heterogeneous protein species as a result of differential glycosylation. The Eg1 and Eg2 display a molecular mass of 60–120 and 45–65 kDa, respectively (Fig. 1b). The sizes are consistent with previous studies (Rose and van Zyl, 2002). The AcEg is secreted as a single protein species of approximately 26 kDa, which is consistent with the predicted unglycosylated mass.

**Fig. 2.**
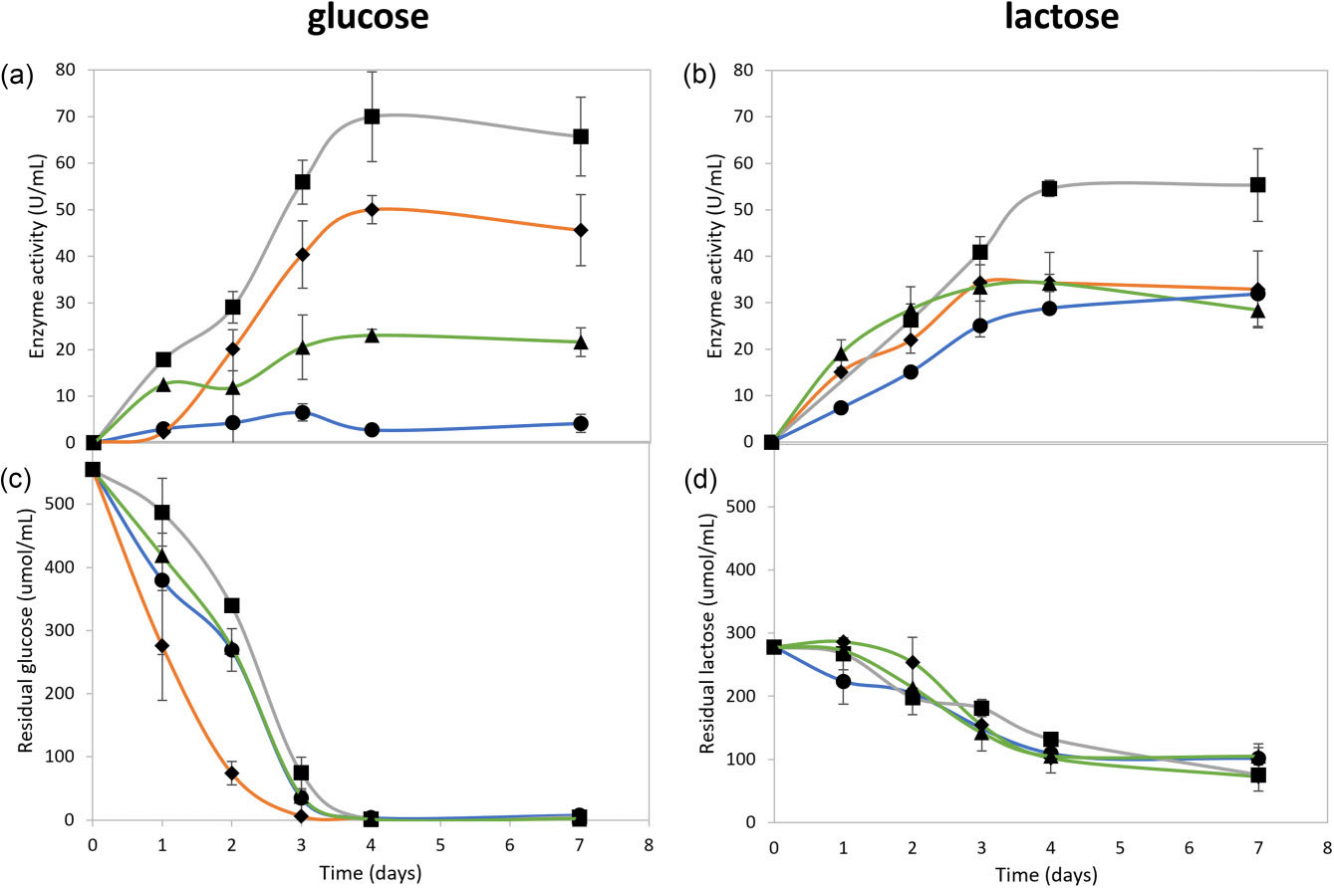
Endoglucanase activity of the recombinant *A. niger* strains on defined media. The extracellular endoglucanase activity (a, b) displayed by -●- *A. niger* D15[GT]PyrG, -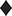
- D15[eg1]PyrG; -■- D15[eg2]PyrG and -▲- D15[aceg]PyrG, as well as the residual carbohydrate source (c, d), was monitored for 7 days in 2 × MM medium containing glucose (a, c) and lactose (b, d), respectively. The values in this figure were obtained using three biological repeats with the error bars indicating the deviation between the biological repeats. *A. niger* D15[GT]PyrG is the reference strain and serves as the negative control.

The recombinant *A. niger* D15[GT]PyrG, D15[egI]PyrG, D15[eg2]PyrG, and D15[aceg]PyrG strains were inoculated to a spore density of 1 × 10^6^ spores/mL in 2×MM (glucose or lactose at equal molar carbon concentrations). Supernatant samples were collected at 24-hr intervals and used to quantify the extracellular endoglucanase activity over 7 days (Fig. 2). The activity was determined at a pH of 4.5 because the pH optima of the recombinant Eg1, Eg2, and AcEg enzymes range from pH 4 to 5 (Rose and van Zyl, 2008). Maximum endoglucanase activity was detected with 4 days of cultivation on glucose, which coincided with the depletion of glucose in the medium (Fig. 2a and c). The *A. niger* D15[egI]PyrG, D15[eg2]PyrG, and D15[aceg]PyrG strains produced a maximum of 50, 70, and 23 U/mL, respectively, whereas the *A. niger* D15[GT]PyrG reference strain produced 6.5 U/mL after 3 days. The activity remained constant for 7 days which is indicative of the stability of the hydrolases under cultivation conditions and the lack of extracellular protease activity.

The *A. niger* D15[eg2]PyrG strain displayed 54 U/mL on lactose (Fig. 2b), which is notably less than with cultivation on glucose (Fig. 2a). The *A. niger* D15[egI]PyrG and D15[aceg]PyrG strains produced around 34 U/mL of endoglucanase activity after 4 days of cultivation (Fig. 2b), whereas the *A. niger* D15[GT]PyrG control strain consistently produced less than 10 U/mL throughout. The residual reducing sugars in the cultivation medium were monitored using the reducing sugar assay (Fig. 2d). The reduced sugar concentration decreased over the first 4 days and was not completely utilised. In this study, a high lactose concentration was used to induce extracellular galactosidase production, but the hydrolysis of the lactose was inadequate to result in glucose accumulation. The *gpd* promoter requires high concentrations of glucose (10% glucose) for maximum expression (van Zyl et al., 2009). The reduced endoglucanase activity on lactose for the *A. niger* D15[egI]PyrG, D15[eg2]PyrG, and D15[aceg]PyrG strains can therefore be ascribed to the lack of glucose in the medium and the strains’ inability to effectively utilise all the carbon in the lactose medium.

**Fig. 3.**
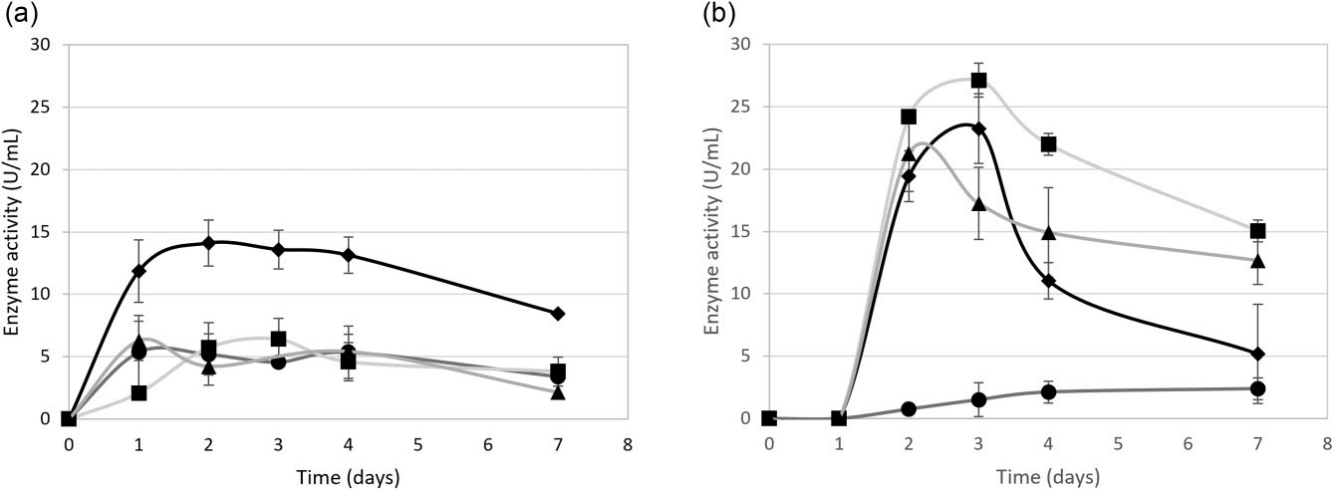
Endoglucanase activity of the recombinant *A. niger* strains on industrial waste media. The extracellular endoglucanase activity displayed by the -●- *A. niger* D15[GT]PyrG, -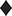
- D15[eg1]PyrG; -■- D15[eg2]PyrG and -▲- D15[aceg]PyrG strains were monitored daily with cultivated in (a) soy whey and (b) bovine whey containing 100 mg/L erythromycin. Standard deviations are representative of three biological replicates. *A. niger* D15[GT]PyrG is the reference strain and serves as the negative control.

The recombinant *A. niger* D15[GT]PyrG, D15[egI]PyrG, D15[eg2]PyrG, and D15[aceg]PyrG strains were cultivated in soy and bovine whey. The extracellular endoglucanase activity was monitored over 7 days. The strains displayed significantly lower levels of activity with cultivation on the soy whey compared to that of bovine whey (Fig. 3a). Extracellular endoglucanase activities of less than 7 U/mL were displayed by the *A. niger* D15[eg2]PyrG and D15[aceg]PyrG strains, similar to that of the D15[GT]PyrG reference strain, whereas the D15[eg1]PyrG displayed a maximum activity of 14 U/mL. The overall low level of activity is directly related to the lack of glucose accumulation for induction of the *gpd* promoter, as explained previously. The soy whey, Avonlac^®^ 282, contained a total sugar concentration of 5% (w/w), resulting in an initial concentration of 0.5% sugar in the cultivation medium. The low carbohydrate concentration is not suited for biomass production, resulting in low titres of secreted enzymes. An increase in the whey concentration results in an increase in viscosity in the medium, making it less ideal for oxygen transfer and fungal cultivation in shake flasks and was therefore not pursued. Eg1 is known to hydrolyse a range of organic polysaccharides, including cellulose and hemicelluloses, as well as chemical substrates (Bailey et al., 1993). This wide substrate range of Eg1 might result in improved hydrolysis of the soy content of the whey, releasing more carbon and nitrogen for metabolic processes, resulting in more Eg1 enzymes being produced.

The *A. niger* D15[eg1]PyrG, D15[eg2]PyrG, D15[aceg]PyrG, and D15[GT]PyrG strains produced maximum endoglucanase activities of 23, 27, 22, and 2 U/mL, respectively with cultivation on 10% bovine whey (Fig. 3b). The *A. niger* D15[eg2]PyrG strain performed better on the bovine whey, which is in contrast to the observation in Fig. 3a. During the strain construction, the gene cassettes are integrated at random throughout the genome, and the effect of the integration is unpredictable. This emphasizes the importance of screening multiple transformants under different cultivation conditions. The general decrease in endoglucanase activity points to the instability of the enzymes in the bovine whey medium after 3 days of cultivation. The effectiveness of the erythromycin started to diminish after 3 days, resulting in bacterial growth and the possible production of bacterial proteases.

**Fig. 4.**
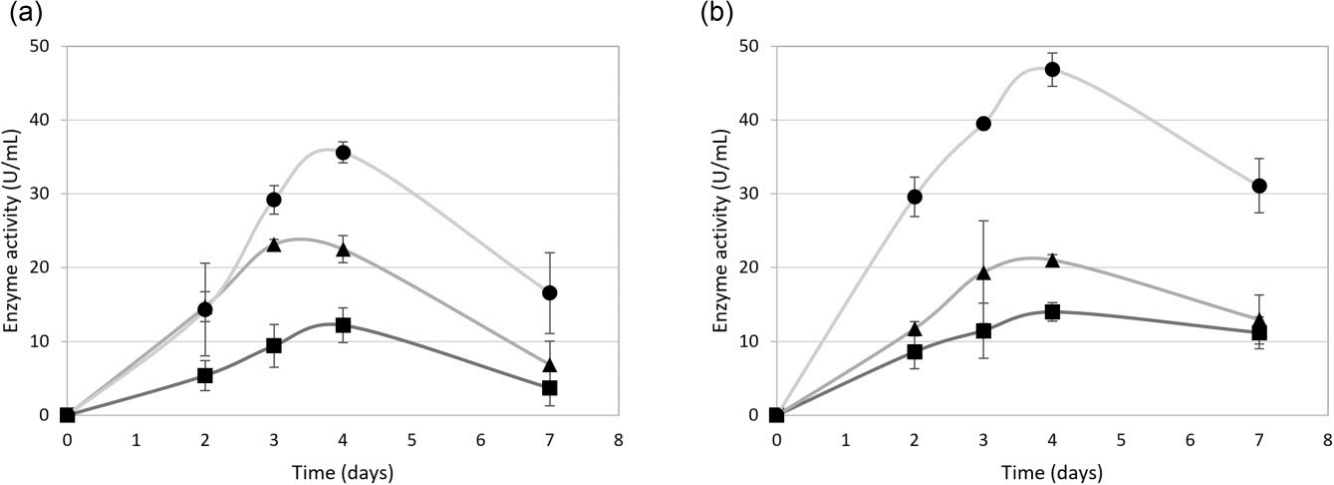
Endoglucanase activity of the best performing recombinant strain. The extracellular endoglucanase activity displayed by the *A. niger* D15[EG2]PyrG strain, was monitored daily with cultivation in -■- 4, -▲- 6, and -●- 10% (a) permeate and (b) whey medium. Standard deviations are representative of three biological repeats.

Bovine whey (Roulou, Stellenbosch, SA) was autoclaved, which resulted in the elimination of bacterial contamination and the coagulation of the remainder of the proteins. The proteins were removed by decanting the liquid whey to simulate lower-grade whey. AspA was added (0.6% NaNO_3_) as an additional nitrogen source after autoclaving. An increase in the permeate or whey concentration increased the extracellular endoglucanase activity displayed by the *A. niger* D15[eg2]PyrG strain (Fig. 4a, b). Maximum activities of 12, 23, and 35 U/mL were obtained on 4, 6, and 10% lower-grade whey medium, respectively after 3–4 days of cultivation. Similarly, 14, 22, and 46 U/mL were obtained in the 4, 6, and 10% whey media, respectively. The proteins in the whey medium can act as a nitrogen source in addition to the exogenous nitrogen added, resulting in higher levels of activity in the whey medium. The parental strain used in this study is a protease-deficient strain, and therefore, has a diminished ability to utilise the extracellular proteins present in the whey. The addition of an external nitrogen source may not be required when a different *A. niger* strain is used as a host. An increase in the whey concentration results in an increase in viscosity in the medium, making it less ideal for oxygen transfer and fungal cultivation in shake flasks and was therefore not pursued. The *A. niger* D15[eg2] strain had previously been cultivated on different forms of potato waste, displaying activities ranging from 20 to 42 U/mL (Rose et al., 2018). The soy whey Avonlac^®^ 282 provides similar results to that of the potato waste but has the advantage that it is easier to transport and can be stored at room temperature.

## Conclusion

Waste products can be used as a substrate for the production of value-added products (valorisation of the waste), which provides economic and environmental benefits for society. With *Aspergillus* being the main industrial enzyme producer and whey being the main waste product produced by the dairy industry, it seems fitting to bring the two together. The whey used in this study supports the growth of the *A. niger* strains without the requirement of additional nutrients. However, it is still advisable to investigate the optimisation of the whey as a cultivation medium or blend the whey with another waste stream to increase the carbohydrate content for increased expression when using the *gpd* promoter. To the best of our knowledge, this is the first report of the evaluation of the *gpd* promoter on lactose as the carbohydrate source. The use of constitutive promoters results in pure enzyme production due to the catabolite repression of the native hydrolases. However, the high levels of glucose required for the induction of the *gpd* promoter are problematic when other carbohydrate sources are used. Therefore, other promoters should be evaluated for use on agricultural waste that contains little to no glucose.

## References

[bib1] Bailey M. J., Siika-aho M., Valkeajärvi A., Penttilä M. E. (1993). Hydrolytic properties of two cellulases of *Trichoderma reesei* expressed in yeast. Biotechnology and Applied Biochemistry, 17(1), 65–76. 8439405

[bib2] Cairns T. C., Barthel L., Meyer V. (2021). Something old, something new: Challenges and developments in *Aspergillus niger* biotechnology. Essays in Biochemistry, 65(2), 213–224. 10.1042/EBC20200139 33955461 PMC8314004

[bib3] Cairns T. C., Nai C., Meyer V. (2018). How a fungus shapes biotechnology: 100 years of *Aspergillus niger* research. Fungal Biology and Biotechnology, 5(1), 13. 10.1186/s40694-018-0054-5 29850025 PMC5966904

[bib4] Fernández-Gutiérrez D., Veillette M., Giroir-Fendler A., Ramirez A. A., Faucheux N., Heitz M. (2017). Biovalorization of saccharides derived from industrial wastes such as whey: A review. Reviews in Environmental Science and Biotechnology, 16(1), 147–174.

[bib5] Food and Agricultural Organisation of the United States (FAO) . (2021). Dairy market review. https://www.fao.org/3/cc1189en/cc1189en.pdf (9 February 2024, date last accessed).

[bib6] Gordon C. L., Khalaj V., Ram A. F. J., Archer D. B., Brookman J. L., Trinci A. P. J., Jeenes D. J., Doonan J. H., Wells B., Punt P. J., van den Hondel C., Robson G. D. (2000). Glucoamylase: Green fluorescent protein fusion to monitor protein secretion in *Aspergillus niger*. Microbiology (Reading, England), 146(2), 415–426. 10708380 10.1099/00221287-146-2-415

[bib7] Laemmli U. K. (1970); Cleavage of structural proteins during the assembly of the head of bacteriophage T4. Nature, 227(5259), 680–685. 10.1038/227680a0 5432063

[bib8] Lech M. (2020). Optimisation of protein-free waste whey supplementation used for the industrial microbiological production of lactic acid. Biochemical Engineering Journal, 157:107531.

[bib9] Li C., Zhou J., Du G., Chen J., Takashi S., Liu S. (2020). Developing *Aspergillus niger* as a cell factory for food enzyme production. Biotechnology Advances, 44: 107630. 10.1016/j.biotechadv.2020.17630 32919011

[bib10] Ling S. O. S., Storms R., Zheng Y., Rodzi M. R. M., Mahadi N. M., Illias R. M., Murad A. M. A., Abu Bakar F. D. (2013). Development of a pyrG mutant of *Aspergillus oryzae* strain S1 as a host for the production of heterologous proteins. The Scientific World Journal. 10.1155/2013/634317 PMC3864154 24381522

[bib11] Miller G. L. (1959). Use of dinitrosalicylic acid reagent for determination of reducing sugar. Analytical Chemistry, 31(3), 426–428. 10.1021/ac60147a030

[bib12] Niu D., Tian X., Mchunu N. P., Jia C., Singh S., Liu X., Prior B. A., Lu F. (2017). Biochemical characterization of three *Aspergillus niger* β-galactosidases. Electronic Journal of Biotechnology, 27:37–43. 10.1016/j.ejbt.2017.03.001

[bib13] O'Connell K. L., Stults J. T. (1997). Identification of mouse liver proteins on two- dimensional electrophoresis gels by matrix-assisted laser desorption /ionization mass spectrometry of in situ enzymatic digests. Electrophoresis, 18(3-4), 349–359. 9150913 10.1002/elps.1150180309

[bib14] Punt P. J., van den Hondel C. (1992). Transformation of filamentous fungi based on hygromycin B and phleomycin resistance markers. Methods in Enzymology, 216:447–457. 1479914 10.1016/0076-6879(92)16041-h

[bib15] Rose S. H., van Zyl W. H. (2002). Constitutive expression of the *trichoderma reesei* β-1, 4-xylanase gene (xyn2) and the β-1, 4-endoglucanase gene (egI) in *Aspergillus niger* in molasses and defined glucose media. Applied Microbiology and Biotechnology, 58:461–468. 11954792 10.1007/s00253-001-0922-3

[bib16] Rose S. H., van Zyl W. H. (2008). Exploitation of *Aspergillus niger* for the heterologous production of cellulases and hemicellulases. The Open Biotechnology Journal, 2(1), 167–175.

[bib17] Rose S. H., Warburg L., Le Roes-Hill M., Khan N., Pletschke B., van Zyl W. H. (2018). Integrated bioremediation and beneficiation of biobased waste streams. In: L. Godfrey, J. F. Görgens, & H. Roman (Eds.), Opportunities for biomass and organic waste valorisation (pp. 108–120). Taylor & Francis Group.

[bib18] Sambrook J., Fritsch E. F., Maniatis T. (1989). Molecular cloning: A laboratory manual. Cold Spring Harbor Laboratory Press

[bib19] van Zyl P. J., Moodley V., Rose S. H., Roth R. L., van Zyl W. H. (2009). Production of the *Aspergillus aculeatus* endo-1,4-*β*-mannanase in *A. niger*. Journal of Industrial Microbiology & Biotechnology, 36(4), 611–617. 10.1007/s10295-009-0551-x 19277742

[bib20] Wiebe M. G., Karandikar A., Robson G. D., Trinci A. P., Candia J. L., Trappe S., Wallis G., Rinas U., Derkx P. M., Madrid S. M., Sisniega H., Faus I., Montijn R., van den Hondel C., Punt P. J. (2001). Production of tissue plasminogen activator (t-PA) in *Aspergillus niger*. Biotechnology and Bioengineering, 76(2), 164–174. 11505386 10.1002/bit.1156

